# Low and High Field Magnetic Resonance for *in Vivo* Analysis of Seeds

**DOI:** 10.3390/ma4081426

**Published:** 2011-08-16

**Authors:** Ljudmilla Borisjuk, Hardy Rolletschek, Johannes Fuchs, Gerd Melkus, Thomas Neuberger

**Affiliations:** 1 Leibniz-Institute of Plant Genetics and Crop Plant Research (IPK), Corrensstraße 3, Gatersleben 06466, Germany; E-Mails: rollet@ipk-gatersleben.de (H.R.); fuchsj@physik.uni-wuerzburg.de (J.F.); 2 Department of Experimental Physics 5 (Biophysics), University of Würzburg, Am Hubland, Würzburg D-97074, Germany; E-Mail: gerd.melkus@ucsf.edu; 3 Department of Radiology and Biomedical Imaging, University of California, 185 Berry Street, San Francisco, CA 94107, USA; 4 Department of Bioengineering, Pennsylvania State University, University Park, PA 16802, USA; E-Mail: tneu@engr.psu.edu

**Keywords:** NMR, MRI, seed quality, crop seed, lipid imaging, sucrose allocation, seed aging, ^13^C

## Abstract

Low field NMR has been successfully used for the evaluation of seed composition and quality, but largely only in crop species. We show here that 1.5T NMR provides a reliable means for analysing the seed lipid fraction present in a wide range of species, where both the seed size and lipid concentration differed by >10 fold. Little use of high field NMR has been made in seed research to date, even though it potentially offers many opportunities for studying seed development, metabolism and storage. Here we demonstrate how 17.5T and 20T NMR can be applied to image seed structure, and analyse lipid and metabolite distribution. We suggest that further technical developments in NMR/MRI will facilitate significant advances in our understanding of seed biology.

## 1. Introduction

Seeds represent a major component of both the human and domestic animal diet, and are increasing in importance as an industrial feedstock. The potential to improve seed quality and/or productivity via biotechnology demands the development of more sophisticated analytical methods to acquire quantitative descriptions of seed composition. Current platforms rely largely on chromatography (HPLC and GC), and various mass spectrometry-based methods applied after tissue dissection and extraction. The primary shortcomings of such analytical methods are that they are both destructive and are unable to discriminate between tissues in heterogeneous materials.

Nuclear magnetic resonance (NMR) technology, in contrast, is capable of performing non-invasive quantitative analyses of metabolites [1,2,3,4]. Optical methods are limited by the poor penetration of light through internal tissue layers (which is a particular problem in the context of the developing seed). However, since NMR devices can acquire relatively precise and robust seed composition data, they have been widely used for the evaluation of seed quality [5,6]. Low field NMR is unable to detect key metabolites such as sugars and amino acids, and does not permit imaging, but this handicap can be overcome by shifting to higher fields and combining it with advanced gradient and radiofrequency resonators as done for example in clinical MRI [7]. Furthermore, NMR of plants has to overcome further difficulties as described in [8].

High field NMR spectroscopy and MR imaging (MRI) have already been used for a variety of seed-related purposes, including the monitoring of water movement [9], imbibition [10] and oil deposition [11,12,13,14,15]. While ^1^H NMR spectroscopy is used for the detection and quantification of major sugars and amino acids, ^1^H chemical shift imaging (CSI) allows in addition to acquire spatial maps from the major metabolites [16,17]. Besides ^1^H NMR, it has been shown that it is possible to use ^13^C NMR spectroscopy to detect ^13^C sucrose [18]. High field ^1^H and ^13^C NMR spectroscopy are particularly well suited for the non invasive quantification of metabolites [8,19,20] and for metabolite profiling [21,22,23]. MRI has been successfully applied to visualize the deposition of lipids in seeds with near cellular resolution [24].

Metabolic and functional imaging is particularly difficult in seeds [8,20]. While NMR pulse-sequences developed in the preclinical and clinical MR field can be adapted for the structural or metabolic imaging of seeds, its exploitation for the inverse detection of ^13^C metabolites requires major adjustments to the software and rigorous optimization. Nevertheless, it is clear that NMR represents a powerful means of non-invasive analysis of seed composition, because it is largely unaffected by variation in seed size, tissue density or tissue layer pigmentation. NMR avoids problems associated with signal penetration or scattering, and generates a high level of spatial resolution. The sensitivity of NMR is less than that achievable using positron emission tomography (PET) [25,26]. However, combining inverse ^13^C/^1^H detection and ^13^C feeding with high field NMR allows for dynamic NMR imaging to be applied to the seed [27]. As yet, the use of high field NMR is not widespread, largely because the cost of the hardware is high and the throughput of the analysis is low. In this paper, we describe some recent applications of NMR spectroscopy and MRI in the area of seed research, which focus on metabolite quantification and imaging.

## 2. Methods

### 2.1. Hardware

A Bruker MQ60 device (Bruker GmbH) was used for the low-field NMR experiments. The instrument has a magnetic field strength of 1.5 T and the probe head is suitable for seeds with a diameter of up to 5 mm and a length of up to 10mm. Both FID and echo acquisition are possible to obtain relaxation measurements of the complete sample. A Bruker 750 MHz WB Avance system (Bruker GmbH) equipped with either a 200 mT/m or a 1 T/m gradient system was used to obtain proton and ^13^C measurements. For proton imaging, a probe head allowing an outer sample diameter of up to 20 mm was used, while the ^13^C experiments were conducted using a double resonant ^1^H/^13^C coil (inner diameter of 5 mm). A standard bore Bruker Avance 850 instrument with a field strength of 20T provided the imaging capability. This scanner comprised a 3 T/m gradient set with an inner diameter of 19 mm. A saddle coil with an inner diameter of 5mm was used for imaging.

### 2.2. Plant Seed Material

*Hordeum vulgare* (Barley cv. Barke) plants were cultivated in a growth chamber under a light/dark regime of 16/8 h at 20/14 °C. Pea plants were cultivated in a greenhouse under a light/dark regime of 16/8 h. NMR measurements were taken from either freshly harvested material, or following its snap-freezing in liquid N_2_ and storage at −80 °C. Stable isotope (^13^C) labelling in barley was performed following Melkus et al. [27]. Each stem was cut 5cm below the ear and placed in ¼ strength Murashige and Skoog medium containing 10 mM glutamine, 10 mM asparagine, 2 mM MES buffer, pH 6.0 and 100 mM UL-^13^C_12_-sucrose (Omicron Biochemicals, South Benol, USA). Artificially aged wheat and barley grains were sampled at the start and at the end of an artificial ageing procedure [28]. The seed materials (*Pisum sativum ‘Erbi’* (Pea), *Trachycarpus fortunei* (Chusan palm), *Poa pratensis* (Kentucky bluegrass), *Robinia pseudoacacia* (black locust), soybean, *Sinapis alba* (white mustard) and *Dactylorhiza fuchsia* (common spotted orchid)) were provided by Charlotte Seal (Royal Botanic Gardens, Kew, UK), and the barley and oat grain and oilseed rape seed by Andreas Börner (IPK Genebank).

### 2.3. Application of MQ60

NMR tubes were loaded with intact dry seeds/grain at 20 °C, and the spin echo signal acquired at 7 ms was averaged over 16 measurements. The signal height of the spin echo is proportional to lipid content, and the proportionality constant was estimated from a range of samples of known lipid content. For this purpose after the NMR analysis, barley and oat grain and oilseed rape seed samples were powdered and extracted to obtain GC measurements of their total lipid content, following Neuberger *et al*. [24,29]. Data on lipid content in seed/grain extracts of other test species were taken from Seal *et al*. [30].

### 2.4. Lipid Mapping of Mature Seeds Using ^1^H MRS-Imaging

The 17.6 T system was used to image naturally aged barley grains, and the 20T system for wheat. For the former, a solenoid resonator with an inner diameter of 2.5 mm was used to image the lipid distribution. The spectra acquired from the whole barley grain confirmed the absence of any MR visible water (data not shown). The analysis of lipid content used a standard three dimensional spin echo sequence with a repetition time (TR) of 0.5 s, an echo time (TE) of 2.4 ms, a field of view (FOV) of 1.0 × 0.5 × 0.5 mm^3^ and a matrix size of 128 × 60 × 60. Due to the low lipid concentrations present, each measurement was repeated six times, resulting in an acquisition time of 3 h. During the reconstruction (Matlab; The MathWorks, Inc., Natick, MA, USA) the data were zero-filled by a factor of two, resulting in a pixel resolution of 39µm. A calibration procedure (described in [24]) was applied to translate the signal intensity of the lipid-specific MR image into lipid content. Signal intensities, as given by pixel number, were recorded in regions of interest (ROIs). The lipid distribution in aged wheat grains was quantified in one experiment using the 850 MHz system. Once again the spectra confirmed the absence of visible water (data not shown). The three dimensional spin echo sequence was TR = 0.8 s, TE = 3.6 ms, FOV = 10 × 4.5 × 4.5 mm^3^, matrix = 282 × 128 × 128, to give a nominal resolution of 35 µm^3^. After zero-filling by a factor of two, the pixel resolution became 17.5 µm isotrop. The data were collected over a relatively long repetition time in order to minimize the effect of any variation in the lipid T1. Similarly, the echo time was reduced to a minimum to minimize the effect of variation in the lipid T2. The experiment was run six times, resulting in a total acquisition time of 21 h 52 min. After the experiment the samples were freeze-dried and their total lipid content measured by GC. Measured MRI signals (normalised ROI values) were plotted against GC values as described by Neuberger *et al*. [24,29].

### 2.5. Localized ^1^H NMR and ^1^H Spectroscopic Imaging on Fresh Harvested Seed

Developing pea seeds (30–280 mg) were analysed with a Bruker 17.6 T widebore spectrometer equipped with a 200 mT/m gradient system and a 15mm diameter birdcage resonator. The local distributions of the metabolites were imaged using a chemical shift imaging sequence [31] with a VAPOR pre-saturation scheme included for water suppression [32]. The parameters were: TR = 1.5 s, TE = 2 ms, NS = 5000, resolution in plane = 200 μm × 200 μm, slice = 250 μm, measurement time = 2 h 5 min). Sucrose (Suc), alanine (Ala) and glutamine (Gln) were quantified using a phantom replacement method, based on work by Soher *et al*. [33]. For the identification and clarification of the NMR visible metabolite signals in the endosperm, a water suppressed localized 2D method (L-COSY, [34]) was applied. Here the parameters were: TR = 2 s, TE = 20 ms, voxel = 1.5 mm^3^, t1-increments = 1024, NA = 1).

### 2.6. Double Resonant ^13^C / ^1^H NMR for Tracing ^13^C Metabolites in Developing Seeds

A gradient enhanced heteronuclear multiple quantum coherence (geHMQC) editing scheme [35] was used for ^13^C sucrose imaging. This sequence detects the protons attached to ^13^C atoms, and is therefore more sensitive than the direct detection of the ^13^C nuclei [36]. The timing of the pulse sequence was adjusted to the ^1^J-coupling constant of Suc (τ = ½ J) (see Figure 6). For complete suppression of higher order coherences of the water resonance, the sequence was extended by including the VAPOR pre-saturation scheme [32]. The sequence parameters were TR = 1.5 s, number of experiments = 7500 (hanning-weighted k-space acquisition), water suppression bandwidth = 500 Hz, MQC-Gradients ratio G1:G2:G3 = 2:2:−1, spatial resolution = 0.25 × 0.25 × 1.5 mm^3^, duration of the full experiment = 3 h 7 min.

## 3. Results and Discussion

### 3.1. Low Field NMR Enables Accurate and Fast Quantification of Seed Lipids in a Wide Range of Species

**Figure 1:**
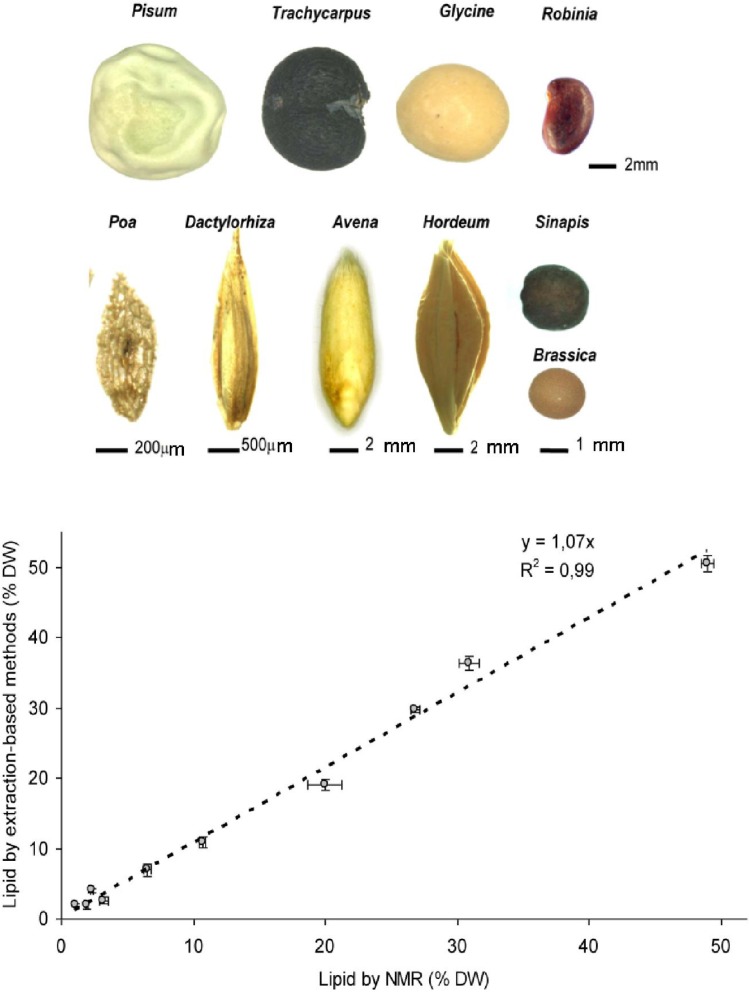
Measurement of seed lipid content using the MQ60 device. (**a**) Correlation of NMR data and conventional assays; (**b**) Seed/grain investigated by NMR were from *Pisum sativum, Trachycarpus fortunei, Glycine max, Robinia pseudoacacia, Brassica napus, Poa pratensis, Dactylorhiza fuchsii, Sinapis alba, Sinapis alba, Avena sativa, Hordeum vulgare*.

The MQ60 NMR spectrophotometer determines the lipid content of the sample (in this case a range of seed/grain) by targeting hydrogen atoms. Minor changes in the behaviour of these atoms in response to changes in the magnetic field are measured by detecting the Hahn echo pulse sequence [37,38]. The relevant protocol has been standardized for some time for the analysis of a number of oilseeds, grains and nuts (ISO 10565). In our hands, the oil content of seeds of highly variable shape (varying in weight by as much as ten fold), pigmentation, surface features (Figure 1a) and oil content (1.5–50.0% on a dry weight (DW) basis) was successfully estimated (Figure 1b). These NMR-based estimates were highly comparable to the those derived from either GC or SFE-CO_2_ analysis, demonstrating that NMR is capable of both the rapid and reliable accurate determination of total lipid content across a wide range of seed type. Several requirements need to be fulfilled before this sort of analysis can be attempted. Initially, the sample must be of a size compatible with that of the glass vials inserted into the NMR device, and the sample should ideally occupy the whole of the vial volume in order to minimize the signal-to-noise ratio. Calibration is essential for each plant species. Both before and during the measurement, the sample must be kept at a constant temperature (above that of the melting point of the seed oil), because the NMR signal amplitude is highly affected by temperature. The amount of seed material required depends somewhat on its lipid content; for example, ~30mg barley grain (lipid content of ~2% DW) was needed, while the requirement for oilseed rape (lipid content ~48% DW) was only 2mg. Finally, it must be recognized that NMR estimations of lipid content derived from intact seed/grain samples tend to be slightly higher than those derived from finely ground samples.

### 3.2. High Field MRI is a Versatile Tool for Exploring Grain Lipid Composition

**Figure 2:**
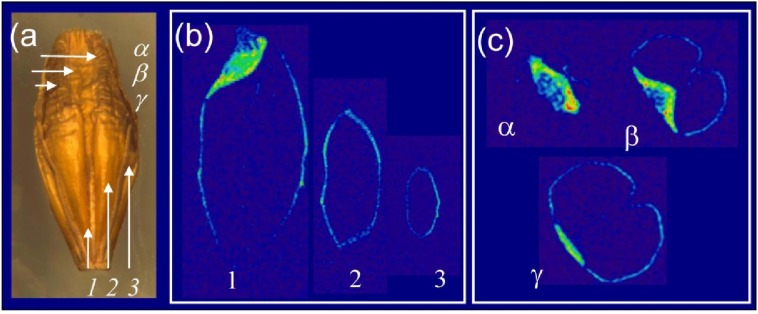
^1^H NMR visualization of lipids in naturally aged barley grains stored for three years. (**a**) Grain used for imaging. Arrows show the placement of the virtual sections (longitudinal 1,2,3 and cross section α, β, γ); Colour-coded visualization of lipid distribution (**b**) along the grain axis (1,2,3) and (**c**) across the cross section (α, β, γ).

It is well understood that seed viability declines during storage, and one of the major causes of this deterioration is thought to be lipid peroxidation (reviewed by McDonald [39]. Pronounced changes in in ^13^C NMR spectra have been documented in western red cedar, most likely resulting from the oxidation of storage oil [22,40]. Studies on natural aging have demonstrated that onion and lettuce seed and rye grain lose viability very rapidly during storage, while viability of pea, lentil, maize and barley is higher [41]. The most extreme seed longevity documented to date has been estimated 1,300 year for old lotus fruit (*Nelumbo nucifera*) [42]. A seed’s ability to retain its viability is likely to depend on its chemical composition and physical structure, which change as the seed ages. Here we have applied NMR to quantify the lipid content of barley grains (Figure 2). Over a three year storage period, the spatial pattern of lipid distribution and lipid content appeared unchanged (data not shown).

**Figure 3:**
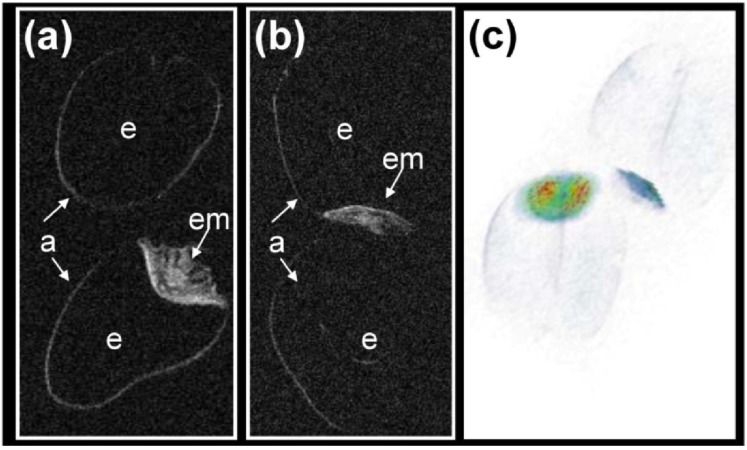
Quantitative NMR based imaging of lipids in living grains at the start (bottom) and end (top) of the artificial aging procedure. (**a**, **b**) Partial 3D representation of lipid distribution in the grain; (**c**) a lipid map showing the fall in embryo oil content in the aged grain (the red colour indicates the maximum lipid content). a: aleurone, e: endosperm, em: embryo.

A number of wheat accessions were also chosen to monitor the effect of artificial ageing. The 3D quantitative MRI assay generated some distinct differences in the lipid signal, particularly between the endosperm and the embryo (Figure 3) and localize lipid depletion/or remains in organ- and tissue-specific manner.

### 3.3. Localized ^1^H NMR and ^1^H Spectroscopic Imaging Allows Assessment of Metabolite Distribution within the Living Seed

Seed development relies on the delivery of sucrose from the maternal tissue to the growing embryo. During early phase of seed development, the embryo is embedded within the endosperm, a structure which is characterized by its high content of soluble metabolites. The endosperm is a key determinant of seed size and quality [17,43]. At least during early developmental stages, endosperm adopt its liquid stage. Conventional studies of liquid endosperm conducted using either HPLC or GCMS involve the destruction of the compartmentation present in the seed, while non-invasive ^1^H MRI both allows the *in vivo* structure to be determined, and identifies the steady state distribution of several critical metabolites. High field localized ^1^H NMR is a well established platform in pre-clinical diagnosis [44,45], but some adjustments were required to adapt the technique for studying the seed. In particular, small NMR coils were introduced in order to improve the signal to noise ratio, since the coils typically used for rodent tissue (inner diameter of 25–40 mm) proved to be too large to obtain an acceptable level of sensitivity in the pea seed. To successfully quantify metabolites, homogeneous volume coils are preferable to surface coils, as their more uniform B1 field distribution avoids the need for the correction of B1 field inhomogeneities. Finally, imaging structural features in the seed required a higher level of resolution than is common in standard clinical imaging set ups. Thus much stronger gradients (200 mT/m and 1 T/m) had to be applied, while retaining sufficiently short echo times to interpret the resulting MR sequences.

**Figure 4:**
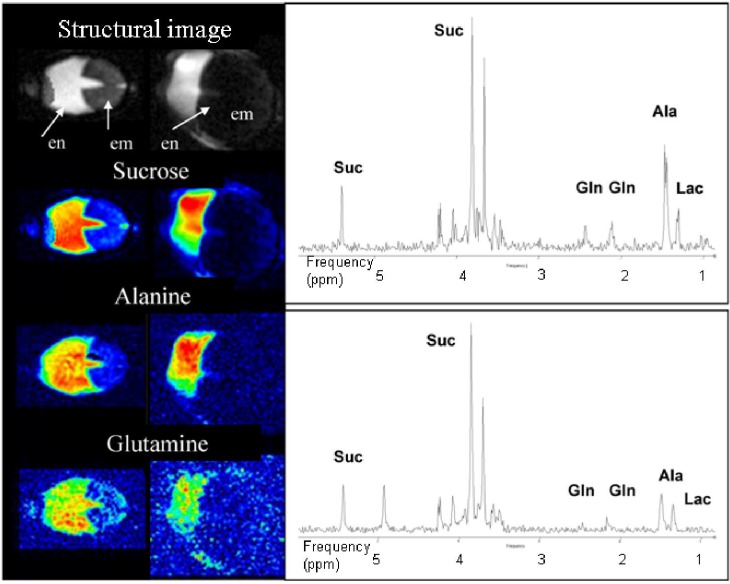
Localized ^1^H NMR and ^1^H spectroscopic imaging used for the assessment of metabolite distribution within an intact living seed. Reference and metabolite images of the pea seed of DW 120 mg (left) and 250 mg (right). Localized spectra of the pea endosperm (right panel). Upper spectrum: 120 mg DW seed, lower spectrum: 250 mg DW seed. A lower alanine (Ala) and glutamine (Gln) signal was obtained from the younger seed.

Figure 4 (left panel) shows a pair of MRI images (FLASH, TR = 60 ms, TE = 3 ms) of the liquid endosperm of a wild type pea seed which had reached a DW of 120 mg (left) and of 250 mg (right). The endosperm is located between the embryo and the maternal testa. Reconstructed images for the distribution of sucrose, alanine and glutamine (Suc, Ala and Gln) in the pea endosperm are shown below the reference images, and typical spectra obtained from the endosperm are given in Figure 4 (right panel). The Suc signal was clearly separated from those originating from Ala (methyl-group) or Gln.

**Figure 5:**
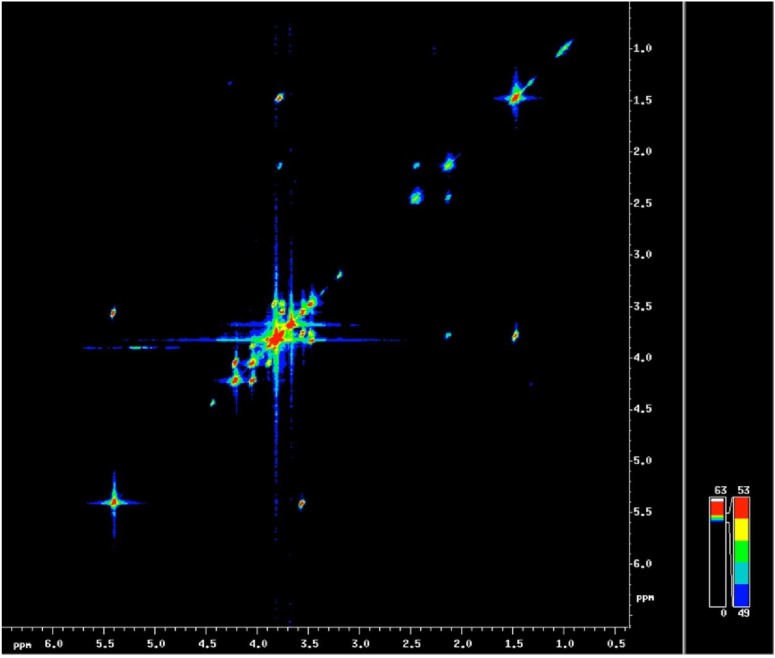
L-COSY spectrum of the endosperm of a pea mutant [17] seed.

2D L-COSY was then used to monitor the occurrence of the various metabolites, taking advantage of cross-peaks generated from J-coupling between the moieties present in the metabolites (Figure 5). NMR was combined with biochemical analysis to characterize the content of the liquid pea endosperm. Metabolite levels were developmental stage specific, in agreement with known events in the endosperm during seed development [17]. The analysis allowed some inferences to be made regarding the metabolic impact of the endosperm in the maternal/filial interaction and the process of seed filling, and finally underlined the importance of the endosperm in the determination of final seed size. Note, however, that the analytical platform can as yet only visualize steady state as opposed to dynamic metabolite levels. The exploration of the dynamics of metabolism is commonly based on isotope feeding experiments [46].

### 3.4. Double Resonant ^1^H /^13^C NMR Allows the Tracing of ^13^C Labelled Metabolites within the Seed

**Figure 6:**
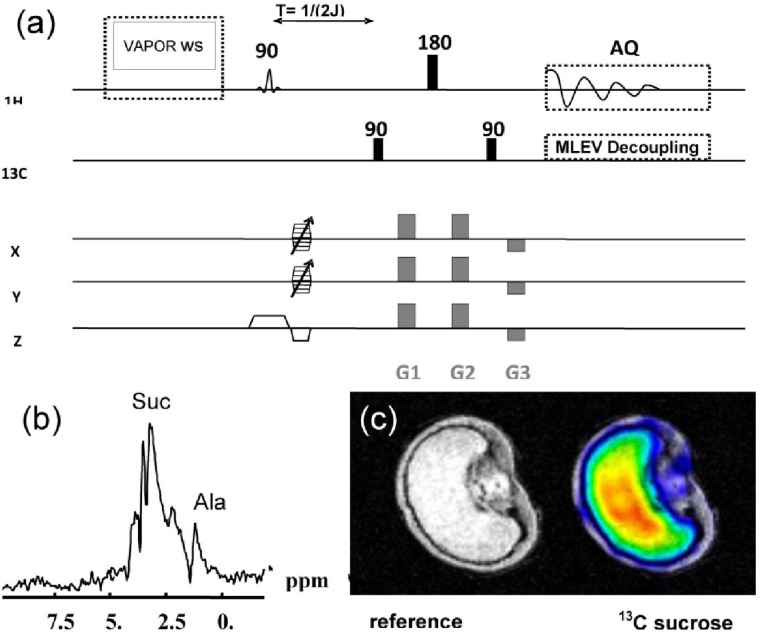
ge-HMQC spectroscopic imaging of a barley grain. (**a**) Pulse sequence with water pre-saturation and ^13^C decoupling; (**b**) ge-HMQC spectrum from the endosperm region; (**c**) reference and metabolic images from ^13^C sucrose after 14h feeding with ^13^C labelled sucrose.

The localisation of ^13^C after during isotope feeding experiments based on tissue dissection. Destructive nature is major disadvantage of feeding experiments, but this can be overcome by the application of ^13^C/^1^H NMR, which generates a spatial resolution in the sub-millimetre range [27]. Here we have demonstrated how the use of a double resonant ^13^C/^1^H coil, in combination with a high magnetic field strength (17.6 T) and feeding with ^13^C enriched Suc, allowed the detection and visualization within the intact seed of not only ^13^C Suc, but also of its metabolized products. Inverse measurement of the ^13^C nucleus over the connected protons was used, a strategy which improves the level of sensitivity compared to what can be achieved by direct ^13^C acquisition techniques [27,36]. The pulse sequence applied for imaging (Figure 6a) produced time-resolved images of ^13^C sucrose uptake (data not shown), as well as also a series of geHMQC spectra from both the endosperm and the testa (Figure 6b). ^13^C Ala, a metabolic product of sucrose, was visualized in a similar way. Thus the distribution of both ^13^C Suc and ^13^C Ala some 14 h after the administration of ^13^C labelled sucrose was visualizable within the intact detached grain (Figure 6c).

## 4. Conclusions

NMR is a powerful analytical tool which allows for the non-destructive quantification and/or visualization of a number of important compounds present in the living seed. In the context of seed research, NMR provides particular advantages where optical and destructive methods are liable to generate artefacts resulting from pigmentation, tissue heterogeneity, sample size variation, *etc*. Combining NMR with other techniques improves the biological interpretation of NMR data. Here we have demonstrated how ^13^C/^1^H NMR can be used to visualize some key metabolites in the pea endosperm, how ^1^H NMR can be applied to study lipid degradation during seed ageing, and how low field pulsed NMR allows for the evaluation of seed composition. We believe that further improvements in NMR spectroscopy and MRI technology will materially advance our understanding of seed metabolism and development.
